# Effect of transcutaneous electrical nerve stimulation on pain, edema, and trismus after surgical removal of impacted third molars: a split-mouth randomized clinical trial

**DOI:** 10.4317/medoral.26193

**Published:** 2023-10-12

**Authors:** Ahad Alizadeh, Aida Karagah, Reza Tabrizi, Leyli Shadman, Azin Arjmand, Maryam Tofangchiha, Romeo Patini

**Affiliations:** 1 Assistant Professor, Medical Microbiology Research Center, Qazvin University of Medical Sciences, Qazvin, Iran; 2 Dental Caries Prevention Research Center, Qazvin University of Medical Sciences, Qazvin, Iran; 3 Assistant Professor, Department of Oral and Maxillofacial Surgery, Qazvin University of Medical Sciences, Qazvin, Iran; 4 Associate Professor, Department of Oral and Maxillofacial Surgery, Shahid Beheshti University of Medical Sciences, Tehran, Iran; 5 Dentist, Student Research Committee, Qazvin University of Medical Sciences, Qazvin, Iran; 6 Dental Caries Prevention Research Center, Qazvin University of Medical Sciences, Qazvin, Iran; 7 Professor, Department of Oral and Maxillofacial Radiology, Qazvin University of Medical Sciences, Qazvin, Iran; 8 Department of Head, Neck and Sense Organs, School of Dentistry, Catholic University of Sacred Heart, Rome, Italy

## Abstract

**Background:**

The transcutaneous electrical nerve stimulation (TENS) stimulus inhibits the activity of nociceptive neurons of the central nervous system. Pain relief is achieved by increasing the pulse amplitude of TENS to induce a non-painful paranesthesia beneath the electrodes. This study aimed to assess the effect of TENS on acute pain, edema, and trismus after surgical removal of impacted third molars.

**Material and Methods:**

This randomized, double blind, split-mouth clinical trial was conducted on 37 patients with bilaterally impacted mandibular third molars. The angle and body of mandible at the site of surgery in one randomly selected quadrant underwent TENS immediately after surgery (50 Hz, 100-µs short pulse, 15 minutes for 6 days). The TENS stimulator device was used in off mode for the placebo quadrant. The pain score (primary outcome) was measured for 7 days postoperatively, and edema and trismus (secondary outcomes) were assessed at 2, 4 and 7 days, postoperatively. The results were analyzed by repeated measures ANOVA using R software (alpha=0.05).

**Results:**

The overall mean pain score was significantly lower in the TENS than the placebo group (*P*<0.05). The number of taken analgesics in the first 3 days was significantly lower in the TENS group (*P*<0.001). Postoperative edema in the TENS group was lower than the placebo group but only the difference was not statistically significant (*P*>.05). The inter-incisal distance, as an index to assess trismus, was not significantly different between the two group at day 2, but it was significantly higher in the TENS group after the second day (*P*<0.001).

**Conclusions:**

TENS effectively decreased pain and trismus following impacted third molar surgery, and may be recommended as a non-pharmaceutical method to relieve postoperative symptoms.

** Key words:**Transcutaneous electric nerve stimulation, acute pain, edema, trismus, molar.

## Introduction

Impacted third molar extraction is a common surgical procedure in dental practice (1). Postoperative pain, edema, and trismus are the most common complications after surgical extraction of third molars, which often occur due to tissue manipulation and surgical trauma (2). Postoperative pain often starts as the effect of local anesthetics disappears, and reaches its peak usually after 12 to 24 hours, postoperatively (3). Maximum edema often occurs 48 hours after surgery, which can affect facial esthetics and social communications of patients; however, it often resolves within 5-7 days (4,5). Trismus has a significant correlation with pain and edema; thus, it reaches its peak on day 2 postoperatively, and gradually resolves by day 7 (1). Postoperative pain and edema often gradually resolve within one week after surgery. However, minimizing these postoperative complications is a priority for both surgeons and patients since they adversely affect the quality of life and cause absenteeism (3,6). Pharmaceutical treatment by administration of non-steroidal anti-inflammatory drugs (NSAIDs), paracetamol, a combination of both, or corticosteroids is the most commonly adopted strategy for the management of mild to severe postoperative pain. However, such medications may fail to alleviate pain in some cases. Also, they are often associated with unwanted local or systemic side effects such as drug interferences or gastrointestinal, hematological, renal, or cardiovascular complications (7-9). Thus, researchers have long been in search of alternative techniques for management of postoperative pain and discomfort. The suggested alternative approaches for this purpose include the application of platelet-rich fibrin, cryotherapy, low-level laser therapy, and transcutaneous electrical nerve stimulation (TENS). These modalities have no significant adverse effect and have been proposed for resolution of pain, edema, and trismus following oral surgical procedures (10,11). TENS is a non-invasive technique with insignificant complications that has shown promising results in reduction of acute orofacial pain (12,13). TENS units are safe, affordable, and available over-the counter. They deliver pulsed alternating current that is applied through the electrodes placed on the skin. The pulse frequency and pulse intensity of TENS are adjusTable and determine its efficacy (14). The mechanism of action of conventional TENS can be explained by the gate control theory discussing that low-intensity and high-frequency stimulation of Aβ afferent fibers with a large diameter leads to segmental inhibition of nociceptive information transfer at the level of dorsal horn (15). The selective stimulation of A-beta fibers (which are large-diameter, non-noxious afferent neurons with low pain threshold) is the rationale behind the application of conventional TENS. The TENS stimulus inhibits the activity of nociceptive neurons of the central nervous system. Pain relief is achieved by increasing the pulse amplitude of TENS to induce a non-painful paranesthesia beneath the electrodes (16). Considering all the above, the aim of this study was to evaluate the effect of TENS on acute pain, edema and trismus following surgical extraction of impacted third molars. The null hypothesis of the study was that TENS would have no significant effect on pain, edema, and trismus following impacted third molar surgery.

## Material and Methods

- Trial design

This split-mouth randomized clinical trial was carried out at the Oral and Maxillofacial Surgery Department of School of Dentistry, Qazvin University of Medical Sciences, from October 2017 to January 2022. The study protocol was approved by the ethics committee of Qazvin University of Medical Sciences (IR.QUMS.REC.1397.332). It was registered by the Iranian Registry of Clinical Trials under the registration number "IRCT20191204045602N1" on the first trial registration date of 24/08/2020. It is confirmed that all experiments were performed in accordance with relevant guidelines and regulations and also informed consent was obtained from all participants or their legal guardians.

- Participants

The patients were selected among those presenting to the Oral and Maxillofacial Surgery Department of School of Dentistry, Qazvin University of Medical Sciences during the aforementioned time period. A total of 37 patients including 21 females and 16 males between 18-35 years with bilaterally impacted mandibular third molars were enrolled.

- Eligibility criteria

Inclusion criteria: The inclusion criteria were presence of bilaterally impacted mandibular third molars with class I difficulty level according to Pell and Gregory classification (all A, B, and C groups) (17) and mesioangular position in both sides, absence of neurological, psychiatric, neuromuscular, endocrine, oral, or maxillofacial conditions, no concurrent treatment with medications such as opioids, antidepressants, or cough suppressants, systemic general health with no underlying systemic condition such as coagulopathy, renal or liver problems, or diabetes mellitus, willingness for participation in the study, attending the follow-up sessions, and filling out the questionnaires.

Exclusion criteria: Patients with pathological mandibular lesions around the impacted teeth, jaw movement limitations, or TMJ problems, those taking sedatives, patients with a history of mandibular angle fracture, and smokers were excluded from the study. All patients signed informed consent forms before the enrollment.

- Surgical procedure 

All surgical procedures were performed by the same oral surgeon. In both the control and intervention groups, the flap incision included an envelope flap with a distal extension. The surgical procedures were performed following administration of a local anesthetic agent for inferior alveolar, lingual, and long buccal nerve blocks for mandibular third molars. A full-thickness mucoperiosteal flap was elevated, and osteotomy was performed with a 1.6-mm round bur connected to a straight surgical handpiece under copious irrigation. The impacted tooth was then completely removed. Odontectomy was performed for cases that needed it. The surgical site was sutured with 3-0 absorbable sutures (braided poly-glycolic acid, reverse cut, 3/8 circle; Pezeshkyaran Amin, Iran).

Surgical extraction of impacted third molar of the contralateral quadrant was conducted after complete resolution of the signs and symptoms of the first surgical procedure, with at least 2-week interval between the two procedures. The duration of surgery was recorded in both procedures and if the procedure at one side took at least 50% longer than the other side, the patient was excluded from the study. The patients were advised to take 500 mg amoxicillin capsules every 8 hours for 7 days; moreover, 400 mg ibuprofen Tablets were prescribed every 8 hours, postoperatively if they experienced severe pain [a visual analog scale (VAS) score ≥5]. The patients were asked to record their VAS pain score and the number of taken analgesics every night. They were also prescribed 0.12% chlorhexidine mouthwash for use twice a day for a period of 10 days. Furthermore, the patients were instructed not to apply ice over the area in order to allow the examiners to assess the effect of TENS on postoperative edema. In the intervention side, patients received TENS (P-620 device of Novin Medical Engineering Co., Iran) in the angle and body of mandible at the site of surgery immediately after surgery. TENS was performed with 50 Hz frequency and 100 µs short pulses for 15 minutes for a total duration of 6 days. In the control side, the electrodes were placed on the skin over the angle and body of mandible at the site of surgery for 15 minutes while the TENS stimulator device was in off mode.

- Variables and primary and secondary outcomes

The use of TENS versus placebo after surgical extraction of impacted mandibular third molars was the predictive variable of this study. The level of pain experienced during 7 days postoperatively was the primary outcome of the study. Postoperative edema and trismus at 2, 4 and 7 days postoperatively were the secondary outcomes. Because of the split-mouth design of the study, the demographic characteristics (age, sex, pain threshold, etc.) of the intervention and control groups were the same.

- Data collection tools

The VAS was used for the assessment of postoperative pain. The patients were asked to express the level of pain they experienced by marking on a 10-cm horizontal VAS with 0 at the left end indicating no pain and 10 at the right end indicating the worst pain possible. The intensity of pain was recorded by patients at bedtime for 7 days, postoperatively. The number of analgesics taken per day was also recorded by the patients themselves. Trismus was assessed by measuring the inter-incisal distance by a ruler and reported in centimeters (cm). Edema was assessed by measuring the distance between the tragus and lip commissure (as horizontal edema), and also the distance between the Gonion and external eye canthus (as vertical edema) by a ruler and reported in centimeters (cm). Both edema and trismus were measured before surgery at 2, 4 and 7 days postoperatively. All measurements were made by an experienced operator blinded to the group allocation of quadrants. The edema coefficient was also calculated using the following formula: Edema coefficient = [(the distance after surgery-the distance before surgery) / the distance before surgery] × 100

- Sample size calculation

The sample size was calculated to be 30 according to a pilot and a previous study assuming alpha error = 0.05, study power = 95% and estimated effect size = 0.4, using G power 3.1.0 software and after taking into account 20% possible dropouts.

- Randomization

Randomization was performed by using sealed envelopes and Random Allocation Software program version 1.0.0 (Isfahan, Iran). Accordingly, the quadrant receiving TENS and the treatment sequence were randomly selected in each patient by a dental assistant not involved in the study, who chose a single sealed envelope every time, and determine the side on which the TENS was to be used.

- Blinding

The examiner, surgeon, patients, and statistician were all blinded to the group allocation of the quadrants. TENS was applied by a trained dental assistant who was not involved in this study.

- Statistical analysis

The results were analyzed by R version 4.1.1 software. Data distribution was evaluated by the Shapiro-Wilk test, which showed normal distribution of data. Thus, repeated measures ANOVA was used to evaluate the effect of group (TENS versus placebo) on the outcomes over time. *P*<0.05 was considered statistically significant.

## Results

**Figure 1:**
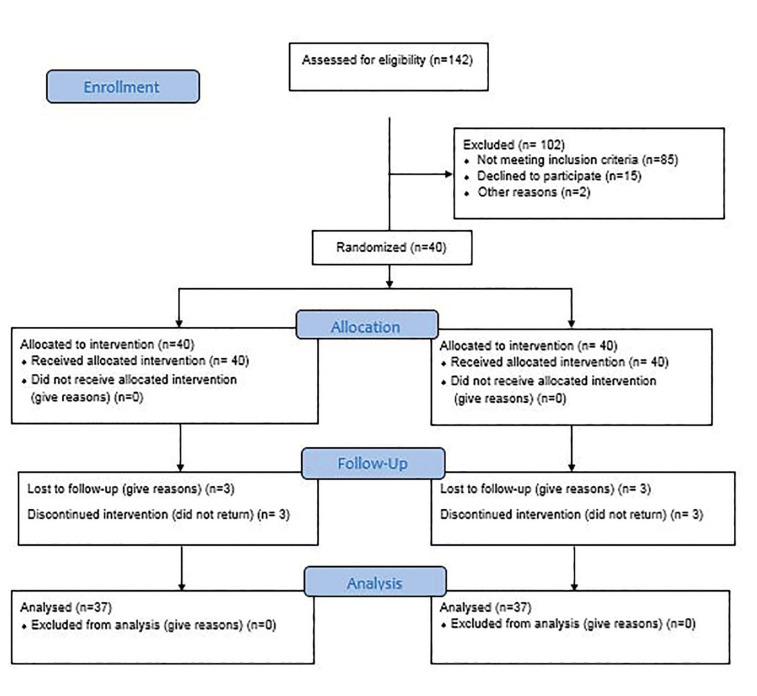
CONSORT flow-diagram of patient selection.

Participant flow: Thirty-seven patients (21 males and 16 females) with a mean age of 23.59 ±4.57 years (range 18-35 years) were evaluated in this study, and a total of 74 impacted third molars were surgically removed. Fig. 1 shows the flow-diagram of patient selection.

Harms: No patients were harmed during the study.

Primary outcome: The pain score in the TENS group was significantly lower than that in the placebo group at all-time points. On day 0 (first night after surgery), the mean pain score in the TENS group was lower than the control group but not significantly (*P*>0.05) (6.43±0.98 in placebo vs 5.21±1.05 in TENS group). However, this difference became statistically significant at 1, 2, 3 days after surgery with a significantly lower pain score in the TENS group (*P*<0.05, Table 1). As expected, a significant reduction in pain score occurred in both groups over time (*P*<0.05). The frequency of analgesic intake was almost the same in the two groups at most time points. The number of analgesics intake at days 2 to 4 (mean differences of 0.91 and 0.37, respectively) was significantly lower in the TENS group (*P*<0.001) but at day 7, there was no statistically significant difference in the number of analgesics taken between the two groups with mean difference of 0.05±0.1 (P≥0.05).

**Figure 2:**
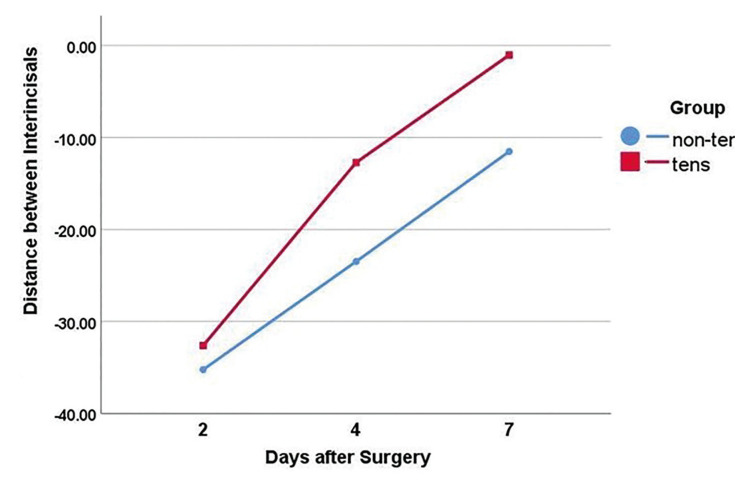
Trend of change in inter-incisal distance in the two groups over time.

Secondary outcomes: The inter-incisal distance (to determine trismus) was not significantly different between the two groups at day 2 (*P*>0.05). However, the inter-incisal distance was significantly greater in the TENS group after day 4 until the end of day 7 (*P*<0.001, Table 2). The inter-incisal distance significantly increased in both the TENS and placebo groups over time (*P*<0.05); nonetheless, this increase was significantly greater in the TENS group than the placebo group at 4 and 7 days (*P* =0.000, Fig. 2). The inter-incisal distance returned to baseline value after 7 days in the TENS group but it was still lower than the baseline value in the placebo group. The distance between tragus and lip commissure was not significantly different in the TENS group at 2, 4 and 7 days after treatment (*P*>0.05). The distance between the gonion and external canthus was not significantly different between the two groups at any time point (Table 3, Table 4).

**Table 1:**
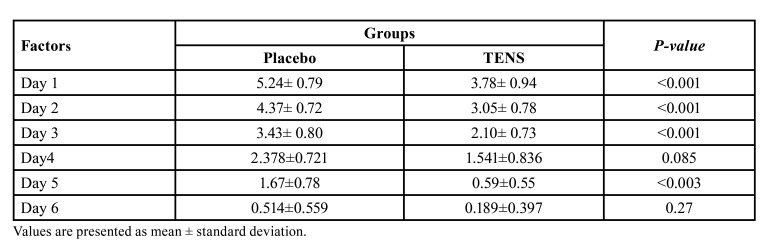
Preoperative and postoperative VAS pain scores of patients in the two groups.

**Table 2:**
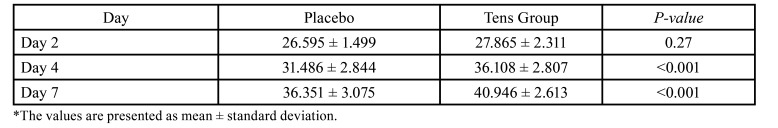
Preoperative and postoperative inter-incisal distance (mm) as an indicator of trismus level.

**Table 3:**
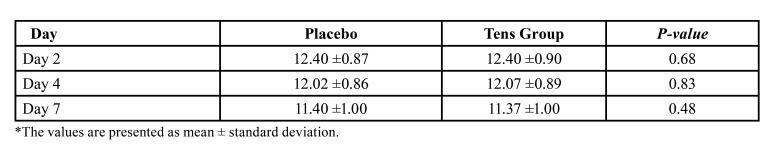
Preoperative and postoperative distance (cm) between the tragus and lip commissure as an indicator of horizontal edema.

**Table 4:**
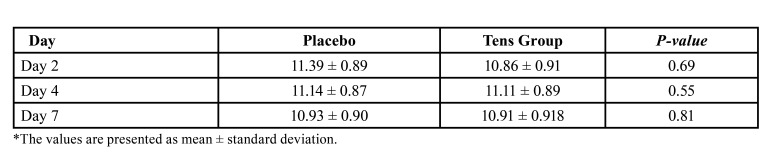
Preoperative and postoperative distance (cm) between the gonion and external canthus as an indicator of vertical edema.

**Figure 3:**
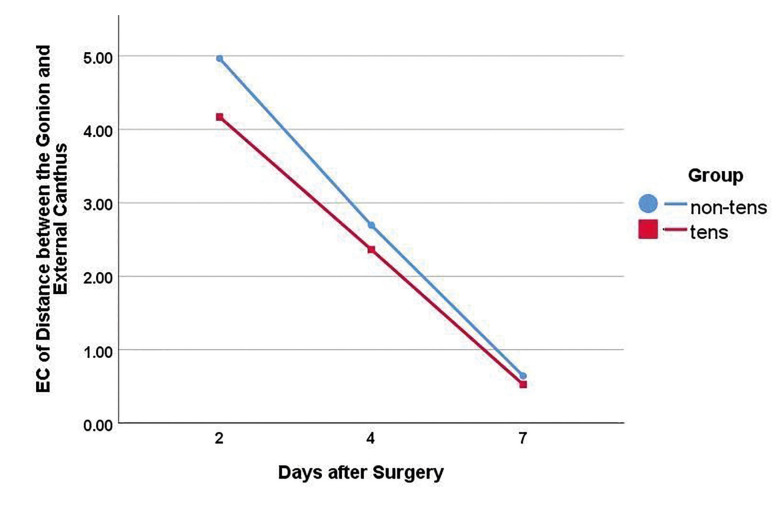
Trend of change in edema coefficient (EC; gonion-external canthus distance) in the two groups over time.

**Figure 4:**
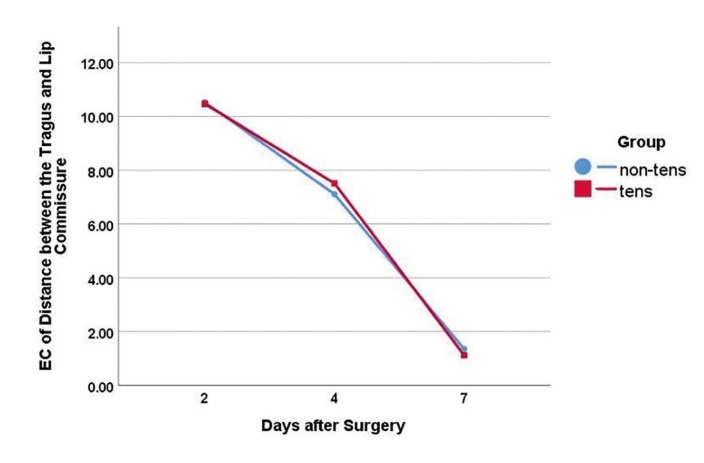
Trend of change in edema coefficient (EC; tragus-commissure distance) in the two groups over time.

Fig. 3 and Fig. 4 present the edema coefficient for the tragus-commissure and gonion external canthus distances (outcome variables) in TENS versus the placebo group (predictive variable) over the study period. The total (collective) postoperative edema coefficient in the TENS group was lower than the placebo group but the difference in edema was not statistically significant (P≤0.05, Table 4). Furthermore, the edema coefficients decreased significantly in both the TENS and placebo groups over time.

## Discussion

This study assessed the effects of TENS on acute pain, edema, and trismus following surgical extraction of impacted third molars. The null hypothesis of the study was that TENS would have no significant effect on pain, edema, and trismus following impacted third molar surgery. The specific aims of the study were: (I) to compare the pain score between the TENS and placebo groups during 7 days after surgery; (II) to compare edema by measuring the tragus-commissure and gonion-external canthus distances at 2, 4 and 7 days after surgery, and (III) to compare trismus between the TENS and placebo groups during 7 days after surgery by measuring the inter-incisal distance. The results showed that postoperative application of TENS over the surgical site significantly decreased acute pain; also, the level trismus was significantly lower in the TENS group compared with the placebo group. Thus, the null hypothesis of the study in pain and trismus was rejected. TENS is a non-invasive modality for pain relief, which is used by placing electrodes on the skin. In the present study, low-intensity stimulation was induced with 50 Hz frequency to stimulate large-diameter A-beta nerve fibers with no effect on smaller fibers (A-delta and C). Resultantly, the patients experience a pleasant paresthesia at the site with a rapid onset which decreases acute pain (18). In addition to analgesia, TENS affects the blood circulation, healing course, and inflammation (19). It affects the afferent nerve fibers, blocks the neural transmission of pain signals (referred to as the gating theory), and induces the release of opioids by the central nervous system; both of the abovementioned mechanisms decrease pain (19). TENS was applied for 15 minutes/day in the present study. The obtained results confirmed the analgesic effects of TENS, as the pain score was significantly lower in the TENS group than the placebo group during the postoperative period. The pain score was measured by using a 10-cm VAS, and the number of analgesics taken daily was also recorded. It was shown that both the VAS pain score and the number of taken analgesics were lower in the TENS group. In the present study, patients were asked to use analgesics only if they needed to at night and after recording their VAS score; therefore, the number of taken analgesics was a true indicator of the level of effectiveness of TENS for postoperative pain control. Although the pain score was significantly lower in the TENS group than the placebo group, the magnitude of daily pain reduction was approximately the same in both groups. As expected, a significant reduction in pain occurred in both groups over time. The frequency of analgesic intake was almost the same in the two groups at most time points. In a study by Çebi, application of TENS decreased the pain score after impacted third molar surgery. It also decreased the need for additional doses of NSAIDs (11).

In the present study, the severity of edema was assessed by measuring the tragus-commissure distance as an indicator of horizontal edema, and gonion-external canthus distance as an indicator of vertical edema. The results showed that horizontal edema was lower in the TENS group than the placebo group at 2, 4 and 7 days after the procedure. However, it was not statistically significant. Vertical edema was lower in the TENS group than the placebo group but it was not statistically significant. Lower degree of facial edema in the TENS group than the placebo group could be attributed to the anti-inflammatory and anti-edematous effects of TENS. Repeated application of TENS for 1 week after surgery probably played a role in lower level of edema in the TENS group in the present study. Nevertheless, the difference was not statistically significant. Similar to VAS scores, the edema coefficients significantly decreased over time in both the TENS and placebo groups. This finding can be attributed to simultaneous improvement of pain and edema, which usually occurs within the first few days after surgery. Interleukin (IL)-6 and tumor necrosis factor-alpha (TNF-α) inflammatory markers are the most efficient indicators of the degree of surgical trauma (20). A meta-analysis revealed the significant efficacy of TENS for reduction of IL6, and TNF-α pro-inflammatory cytokines. Also, opioid release has been reported as one mechanism of action of TENS. Opioids affect the central nervous system by activating the sympathetic or parasympathetic nervous systems (19). Enhanced sympathetic activity elevates the level of catecholamines circulating in the blood, and further decreases the level of pro-inflammatory and anti-inflammatory cytokines such as IL-10 (20). Trismus which refers to difficulty in jaw opening as the result of muscle spasm is another postoperative complication of impacted third molar surgery (21). The main causes of postoperative trismus include flap elevation beyond the external oblique ridge, repeated stimulation of the medial pterygoid muscle by inferior alveolar nerve block, and low-grade infection after local anesthesia, among others (22). By decreasing edema, TENS can decrease the thickness of the masseter muscle, and subsequently decrease the muscle force and pain as such (23). Moreover, the tension along the muscle fiber membrane can be relaxed by TENS (24). Modulation of pain by TENS along with the anti-inflammatory effect of prescribed antibiotics may alleviate the spasm as well (25). Olaogun *et al*. compared the efficacy of TENS, cryotherapy, and placebo for reduction of pain, trismus, and edema following third molar extraction surgery. TENS and cryotherapy were performed for 6 days for 20 minutes/day, and the level of pain, edema, and trismus was measured on days 1 and 6. The results showed higher efficacy of TENS and cryotherapy than the placebo for resolution of pain and trismus following oral surgical procedures. However, TENS and cryotherapy had no significant effect on edema (19). Edema is often maximized at 2 days after surgery, and then gradually resolves by day 7 (4,5). The conventional TENS was used in the present study which appears not to be strong enough to induce muscle contraction and aid in venous return. Also, the period of study was 6 days for each patient; while, the speed of resolution of edema may vary in different patients. Eshaghpour *et al*. used low level laser therapy to decrease pain and edema following third molar extraction surgery and showed that both TENS and low level laser therapy decreased the severity of pain and edema during the first days after third molar extraction surgery, which was in agreement with the present findings in pain. Although both modalities are noninvasive and non-pharmaceutical, laser therapy needs to be performed both intra- and extra-orally (26). The main limitation of this study was the difficulty to select patients with symmetrically impacted mandibular third molars in their right and left quadrants. Therefore, in this study, the therapeutic effects of low frequencies (<10 Hz), high frequencies (>100 Hz), or mixed (high and low) frequencies of stimulation were not evaluated individually.

## Conclusions

Generally, symptoms are most severe in early days following surgery, however, the pain, and trismus significantly decreased in both groups after four days in the present study. TENS effectively decreased pain following impacted third molar surgery. Since the purpose of using TENS is to reduce these complications in the first days after surgery in patients who are unable or unwilling to take analgesics, it may be a suiTable option for such patients. Also, TENS relieved trismus after surgery, which cannot be achieved by analgesics.
